# Supplementary figures and images for: Biodistribution PET/CT Study of Hemoglobin-DFO-89Zr Complex in Healthy and Lung Tumor-Bearing Mice
Source: Int J Mol Sci. 2020 Jul 15;21(14):4991. doi: 10.3390/ijms21144991 (PMC7404105; doi:10.3390/ijms21144991)

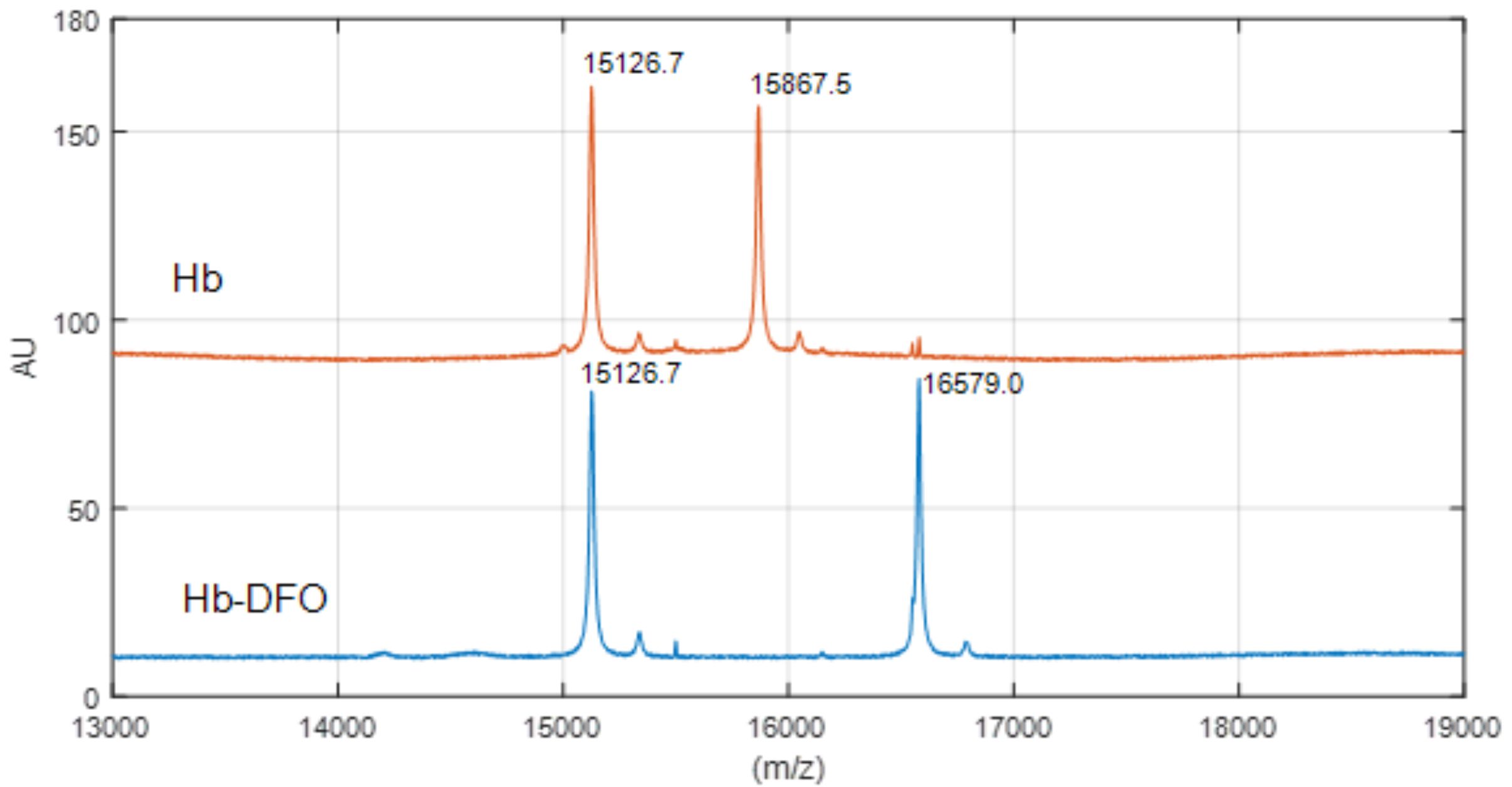

Supplement: Supplementary file 1 [file ijms-21-04991-s001.zip › Fig. S1.jpg]

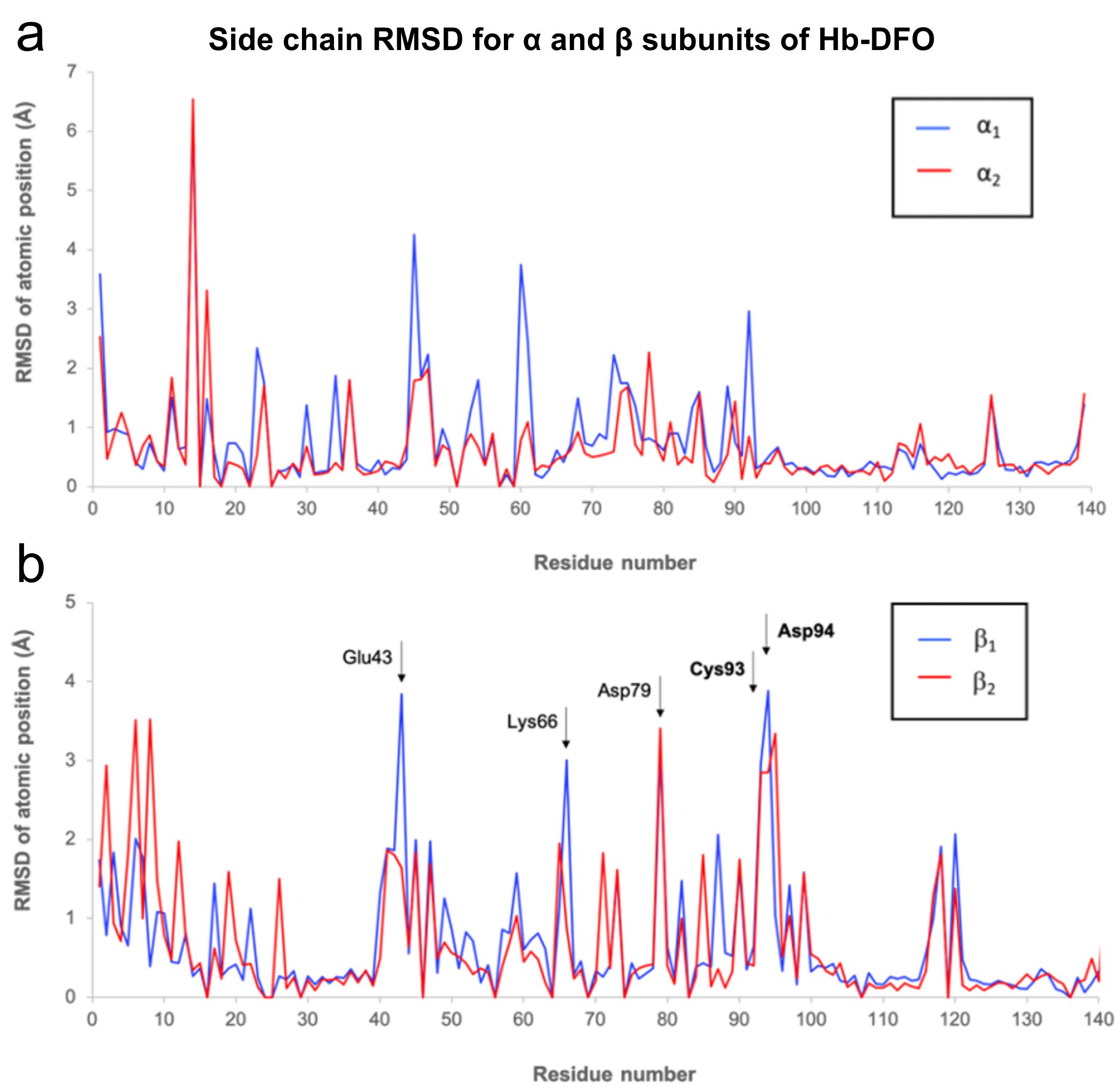

Supplement: Supplementary file 1 [file ijms-21-04991-s001.zip › Fig. S2.jpg]

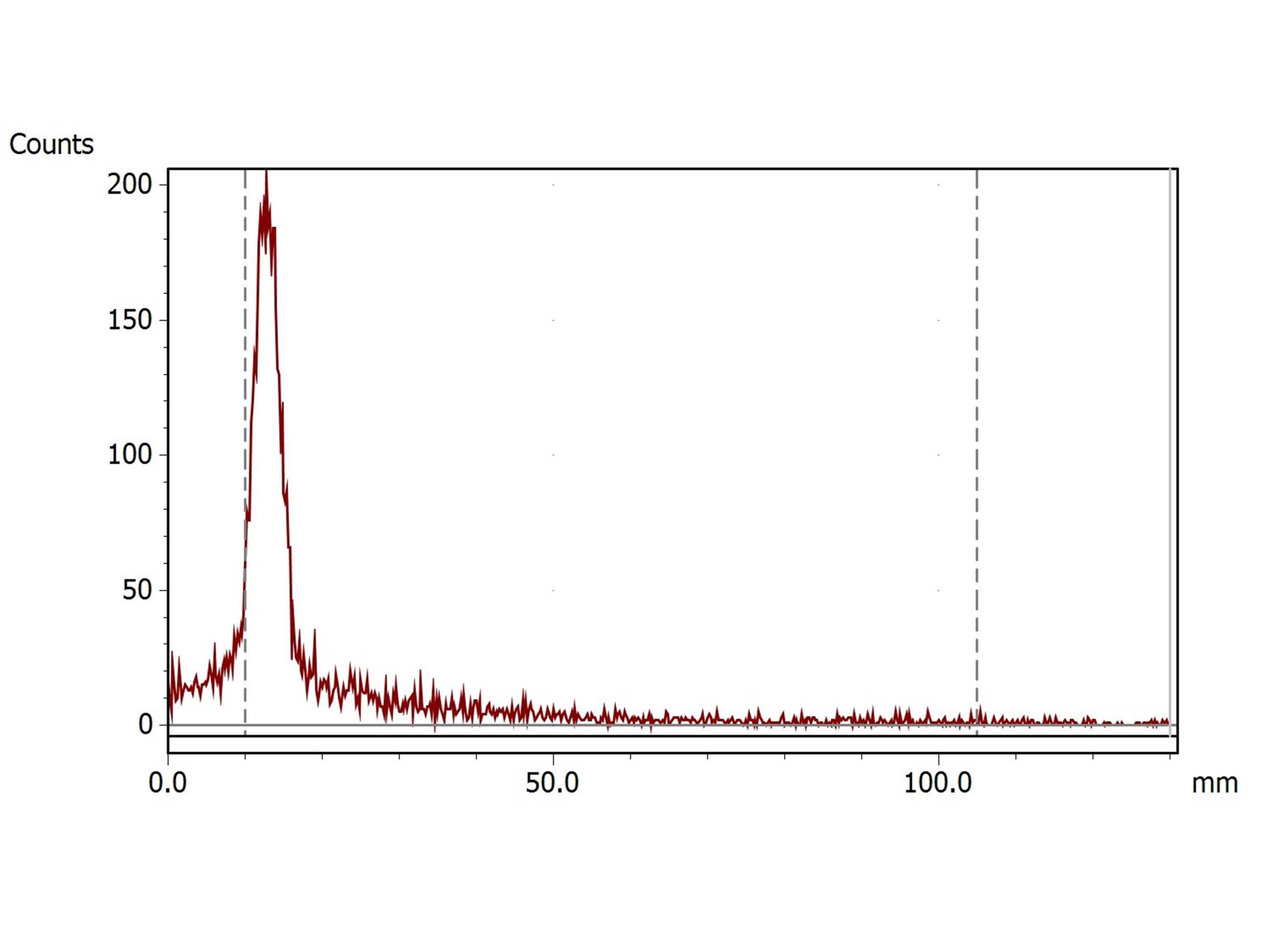

Supplement: Supplementary file 1 [file ijms-21-04991-s001.zip › Fig. S3.jpg]
